# Hydrogels With Tunable Mechanical Properties Based on Photocleavable Proteins

**DOI:** 10.3389/fchem.2020.00007

**Published:** 2020-01-28

**Authors:** Dongfang Xiang, Xin Wu, Wei Cao, Bin Xue, Meng Qin, Yi Cao, Wei Wang

**Affiliations:** ^1^Key Laboratory of Intelligent Optical Sensing and Integration, National Laboratory of Solid State Microstructure, and Department of Physics, Collaborative Innovation Center of Advanced Microstructures, Nanjing University, Nanjing, China; ^2^Shenzhen Research Institute of Nanjing University, Shenzhen, China; ^3^Chemistry and Biomedicine Innovation Center, Nanjing University, Nanjing, China; ^4^Institute of Brain Science, Nanjing University, Nanjing, China

**Keywords:** photocleavable protein, tunable mechanical properties, hydrogel, spatially control, artificial extracellular matrices, on-demand, drug delivery

## Abstract

Hydrogels with photo-responsive mechanical properties have found broad biomedical applications, including delivering bioactive molecules, cell culture, biosensing, and tissue engineering. Here, using a photocleavable protein, PhoCl, as the crosslinker we engineer two types of poly(ethylene glycol) hydrogels whose mechanical stability can be weakened or strengthened, respectively, upon visible light illumination. In the photo weakening hydrogels, photocleavage leads to rupture of the protein crosslinkers, and decrease of the mechanical properties of the hydrogels. In contrast, in the photo strengthening hydrogels, by properly choosing the crosslinking positions, photocleavage does not rupture the crosslinking sites but exposes additional cryptical reactive cysteine residues. When reacting with extra maleimide groups in the hydrogel network, the mechanical properties of the hydrogels can be enhanced upon light illumination. Our study indicates that photocleavable proteins could provide more designing possibilities than the small-molecule counterparts. A proof-of-principle demonstration of spatially controlling the mechanical properties of hydrogels was also provided.

## Introduction

Photo-switchable proteins have been widely used in many fields, including super resolution imaging, optogenetics, fluorescent sensors, and recently as switchable crosslinkers for the engineering of photo-responsive hydrogels (Christie, 2007; Rogan and Roth, 2011; Shcherbakova et al., 2012, 2015; Nienhaus and Nienhaus, 2014; Guntas et al., 2015; Koetting et al., 2015; Niopek et al., 2016; Coquelle et al., 2018). Comparing to other environment-responsive hydrogels, photo-responsive hydrogels have drawn considerable interest (Murphy et al., 2007; Yuan et al., 2008; Gillette et al., 2010; Patterson and Hubbell, 2010; Davis et al., 2011; Yoshikawa et al., 2011; Burdick and Murphy, 2012; Stowers et al., 2015; Abdeen et al., 2016; Han and Lin, 2016), because their mechanical properties can be remotely and non-invasively controlled upon light illumination (Kloxin et al., 2009; Guvendiren and Burdick, 2012; Rosales et al., 2015; Kim et al., 2019; Nowak and Ravoo, 2019; Wu et al., 2019). Generally, most photo-responsive hydrogels are based on the switch between oligomeric and monomeric states of proteins upon light illumination. For example, the reversible change between tetramers and monomers of Dronpa145N can lead to photo-controlled gel-sol transition or reversible change of the stiffness of the hydrogels (Lyu et al., 2017; Xin et al., 2017). Similarly, the switch between dimeric and monomeric states of cyanobacterial phytochrome 1 can lead to the reversible softening and strengthening of the hydrogels, which allowed dynamic control of the migration of immune cells and mechanotransduction of stem cells (Hörner et al., 2019). The change between dimeric and monomeric states of UVR8-1 led to the design of hydrogels for photo-controlled protein delivery and cell separation (Zhang et al., 2015). Green light induced tetrameric to monomeric transition of the C-terminal adenosylcobalamin binding domain (CarHC) can cause the dissolution of the hydrogels for the stem cell/protein release (Wang et al., 2017). Recently, it was shown that the photo-induced conformational change of LOV2 domains can be used to dynamically control the mechanical properties of the corresponding proteins (Liu et al., 2018). This method is unique in that it does not involve oligomeric photo-switchable proteins but utilize the change of crosslinker length. It is desirable to explore new operation mode of photo-controllable hydrogels based on photo-responsive proteins.

Here, we report two new types of photo-controllable hydrogels based on photo cleavable fluorescent protein PhoCl. PhoCl protein was engineered by Campbell group in 2013 (Zhang et al., 2017). Upon the illumination of violet light (~400 nm), the chromophore undergoes a β-elimination reaction, leading to the cleavage of the polypeptide backbone. The two fragments of the protein then spontaneously dissociate and become unstructured. We envisioned that this reaction can lead to two effects: (i) photo-induced cleavage of the PhoCl and (ii) exposure of reactive sidechains inside the barrel structure of the protein. Based on these two effects, we design two types of hydrogels whose mechanical stability can be lowered or elevated upon photo illumination, respectively. We anticipate that these hydrogels can be used as artificial extracellular matrices for cell culture and as cargos for on-demand drug delivery.

## Materials and Methods

### Gene Construction

PhoCl and its sequences have been reported. The gene encoding Pho-Weak and Pho-Strong proteins (Supplementary Figure 1) were purchased from Genscript and cloned into the pQE80L vector between *BamH*I and *Kpn*I restriction enzyme sites. To produce the pQE80L::Pho-Weak-(GB1)_2_ and pQE80l::Pho-Strong-(GB1)_2_, two GB1 (MDTYKLILNGKTLKGETTTEAVDAATAEKVFKQYANDNGVDGEWTYDDATKTFTVTERS) fragments cut by *BamH*I and *Kpn*I restriction enzymes were inserted between *Bgl*II and *Kpn*I sites of pQE80L::Pho-Weak and pQE80l::Pho-Strong, respectively. All genes bear a N-terminal 6× His tag sequence from the pQE80L vector to facilitate affinity purification of expressed proteins.

### Protein Expression and Purification

All proteins were expressed in *Escherichia coli* strain BL21. The bacterial cells were grown at 37°C in LB medium supplemented with 100 μg·mL^−1^ ampicillin to an OD600 of 0.5 followed by adding 240 μg·mL^−1^ isopropyl β-D-1-thiogalactopyranoside to induce protein expression overnight at 25°C. The *E. coli* cells were harvested by centrifugation and the 6 × His-tagged proteins were purified using Ni^2+^-NTA protein resin (GE healthcare, Shanghai, China). The purified proteins were dialyzed against phosphate-buffered saline (PBS) buffer (10 mM, pH7.4, containing 137 mM NaCl and 2.7 mM KCl) and stored at 4°C before use.

### Characterization of PhoCl Mutants

All the photoconversion for ultraviolet-visual (UV-Vis) spectroscopy measurements and fluorescence spectra measurements were performed with a light emitting diode (LED) lamp (M405L3c, THORLABS, USA). Absorbance spectra were acquired with V-550 UV/VIS Spectrophotometer (JASCO). Fluorescence spectra were acquired with FP-6500 Spectrofluorometer (JASCO). Circular dichroism spectra were acquired with J-815 CD Spectrometer (JASCO) at room temperature. MALDI-TOF Mass spectra of PhoCl mutants before and after N-methylmaleimide labeling were recorded using an autoflex TOF/TOF mass spectrometer (Bruker Daltonics).

### Preparation of PhoCl-PEG Hydrogels

To prepare Pho-Weak-PEG hydrogels, the Pho-Weak and 4-armed-PEG-Mal (Mw: 20 k, Laysan Bio, Inc) were first dissolved in PBS buffer and then were quickly mixed at a molar ratio of 2:1. To prepare Pho-Strong-PEG hydrogels, the Pho-Strong and 4-armed-PEG-Mal solutions were mixed at a molar ratio of 1:2. After preparing the gels at room temperature, they were incubated at 37°C for at least 2 h in dark conditions to ensure the hydrogels were stable.

### Characterization of PhoCl-PEG Hydrogels

The photoconversion for rheology measurements was performed with the same LED lamp (M405L3c, THORLABS, USA) as the spectral experiments. Light intensity was 468.6 mW·cm^−2^ in all experiments as measured by an optical power meter (PM100USB, THORLABS, Gemany) with thermal power sensor (S405C, THORLABS, Gemany). The rheological properties of PhoCl-PEG hydrogels measurements were carried out using a HAAKE RheoStress 6000 (Thermo Scientific) at 25°C.

## Results and Discussion

### Design of Photo-Controllable Hydrogels

**Figure 1:**
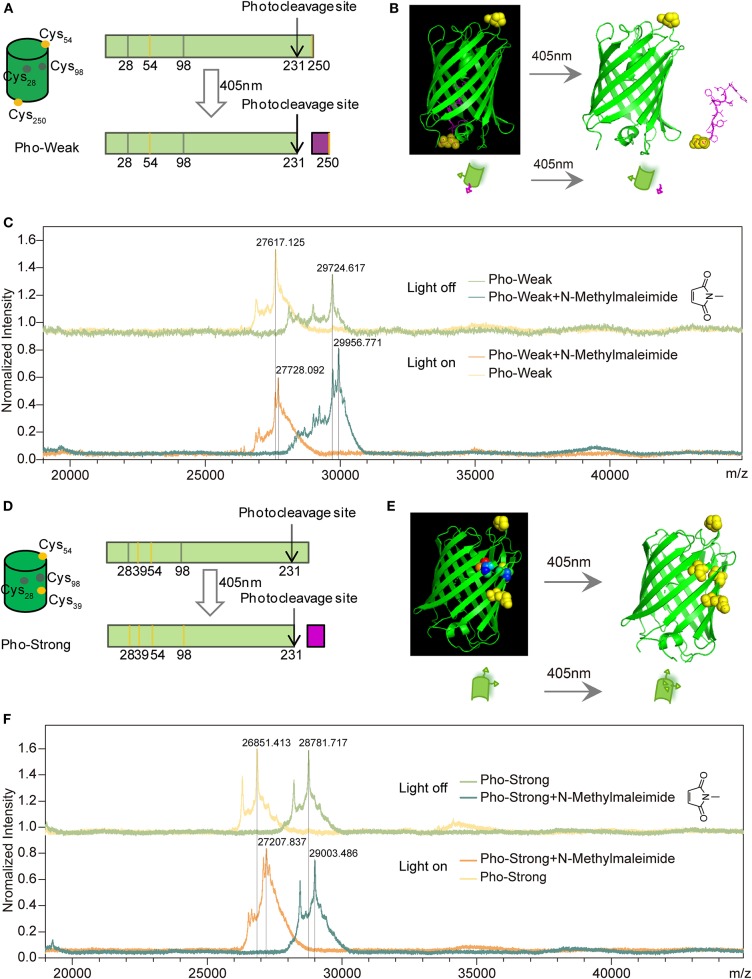
Design of PhoCl mutants. **(A)** Schematic of the design of Pho-Weak. Two solvent exposed cysteines at 54 and 250 positions (colored in yellow) were introduced to wild type PhoCl for crosslinking with maleimide groups in PEG to form hydrogels. The photocleavage reaction leads to the rupture of the linkage between the two crosslinking sites. **(B)** Schematic illustration of the dissociation process. **(C)** MALDI-TOF before and after Pho-Weak photocleavage excited at 405 nm. Two N-Methylmaleimide (Mw:111.1) reacted with Pho-Weak in the dark and the rupture of the linkage between the two crosslinking sites. **(D)** Schematic of the design of Pho-Strong. Photocleavage does not break the linkage between the two crosslinking sites (Cys39 and Cys 54) but expose two cryptic cysteine residues (Cys28 and Cys98, colored in gray). **(E)** Schematic illustration of the light-induced exposure of additional cysteine residues. **(F)** MALDI-TOF of Pho-Strong. Two N-Methylmaleimide crosslinked with Pho-Strong in the dark and more crosslinking sites were formed due to the light-induced exposure of additional cysteine residues.

In wild-type PhoCl, there are two buried (Cys28 and Cys98) but no solvent exposed cysteine residues. Photocleavage takes place at His231 (Supplementary Figure 1), which is part of the chromophore after maturation. In order to incorporate the PhoCl proteins into the hydrogel network, we designed two PhoCl mutants named as Pho-Weak and Pho-Strong proteins, respectively. Each of these two mutants contains two additional solvent exposed cysteine residues that are ready to react with maleimide containing polyethylene glycol (PEG) to formed hydrogels (named as PhoCl-PEG hydrogels hereafter). In the Pho-Weak mutant, one of the cysteine residues is at the 54th position, while the other is attached to the C-terminal end (Figure 1A and Supplementary Figure 1). Photocleavage of Pho-Weak proteins causes the rupture of the sequence between the two cysteine residues (Figure 1B). To confirm this design, we used MALDI-TOF mass spectroscopy to characterize the reactivity of the cysteine residues and the photolysis products. A small maleimide containing molecule, N-methylmaleimide, was used for labeling and the mass gain after reaction is 111.1 Da (Δ). The MALDI-TOF experiments confirmed that the solvent exposed cysteines of Pho-Weak can successfully react with N-methylmaleimide leading to a mass gain of ~2 × Δ (Figure 1C). After photocleavage, the increase of the mass of the larger fragment was ~1 × Δ, suggesting that the cleavage site was in between the two labeled cysteine residues. In the Pho-Strong mutant, the two solvent exposed cysteine residues are introduced at the 39 and 54th positions (Figure 1D and Supplementary Figure 1). Even after photocleavage, the two cysteine residues are still attached to the same fragment (Figure 1E). Similarly, this design was confirmed by the MALDI-TOF experiments (Figure 1F). Before photolysis, the mass gain after labeling was ~2 × Δ; after photolysis and further reacting with extra N-methylmaleimide, the mass gain was ~3.2 × Δ, indicating that some of the originally buried cysteine residues were exposed and reacted with maleimide. Therefore, the crosslinking density increased after photocleavage of Pho-Strong mutants.

**Figure 2:**
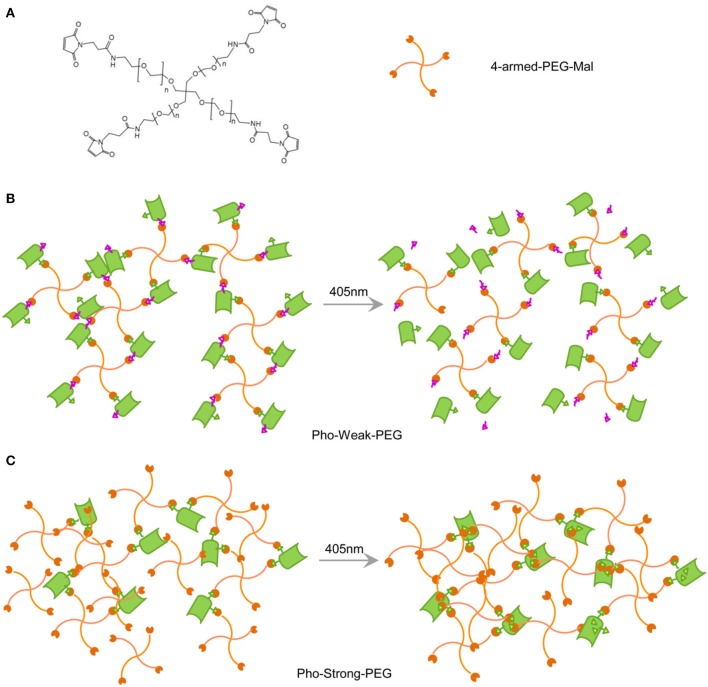
Design of PhoCl-PEG hydrogels. **(A)** Chemical structure of 4-armed-PEG-Mal. The maleimide-terminated end of each arm can react with thiol groups of cysteines when they are solvent exposed. **(B)** Schematic illustration of the Pho-Strong-PEG hydrogel in dark (left) and the photo-switching process of the hydrogel (right). **(C)** Schematic illustration of the Pho-Weak-PEG hydrogel in dark (left) and the photo-switching process of the hydrogel (right).

The hydrogels whose mechanical properties can be enhanced and decreased upon light illumination were prepared based on Pho-Weak and Pho-Strong proteins, respectively. The solvent exposed thiol groups of the two proteins can react with maleimide functionalized 4-armed polyethylene glycol (4-armed-PEG-Mal) to form hydrogel networks (Figure 2A). In the photo-weakening hydrogel, the ratio of maleimide and solvent exposed thiol groups is 1:1 and there were no additional maleimide groups available to react with the freshly exposed Cys28 and Cys98 (Figure 2B). Photocleavage reaction leads to the decrease of the mechanical stability or even gel-sol transition of the corresponding hydrogels. In the photo-strengthening hydrogel, the ratio of maleimide and the total thiol groups (including both the solvent exposed and the buried ones) is 1:1. Photocleavage of Pho-Strong does not cause the decrease of crosslinking density, as the two photocleavage site is outside the crosslinking sequence (Figure 2A). Moreover, because there are additional maleimide groups available from the hydrogel network, new crosslinking sites are readily formed with the exposed cysteine residues (Cys28 and Cys98) after the dissociation of the cleaved fragment (Figure 2C). This results in the elevation of the mechanical stability of the hydrogels after photocleavage. In order to quantify the amount of thiol reacted with maleimide in the hydrogel network, we used a model system by replacing 4-armed PEG-Mal with linear PEG-Mal (only a single maleimide group at the end of each PEG polymer) and analyzed the amount of reacted proteins based on the SDS-PAGE (Supplementary Figure 2). The yields for the Pho-Weak and Pho-Strong were 54 and 65%, respectively. Note that, some cysteine residues may have already formed disulfide bonds before reacting with maleimide groups in the hydrogels, which can also serve as the crosslinks.

### Characterization of the Photocleavage of PhoCl Mutants

**Figure 3:**
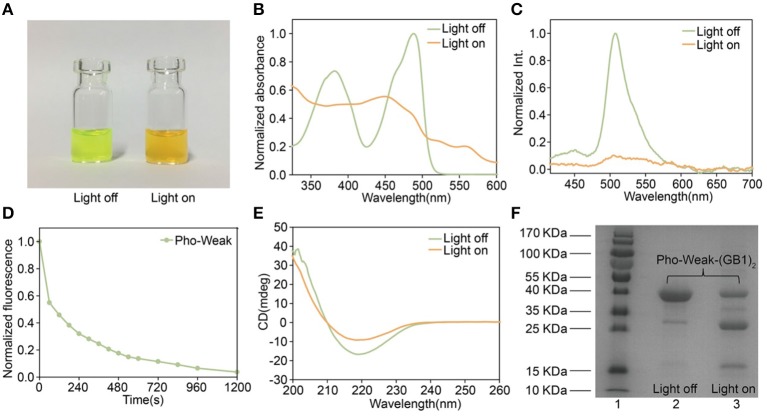
Characterization of Pho-Weak. **(A)** Photographs of Pho-Weak solutions before and after light illumination. **(B)** UV–vis absorbance changes after photocleavage. **(C)** Fluorescence spectra before and after photocleavage excited at 405 nm. **(D)** Kinetics of dissociation, as determined by the decrease of green fluorescence after illumination. **(E)** Circular dichroism spectra before and after photocleavage. **(F)** SDS-PAGE analysis of Pho-Weak-(GB1)_2_ before and after photocleavage. The band at ~ 13 kDa corresponds to the fragment containing (GB1)_2_.

**Figure 4:**
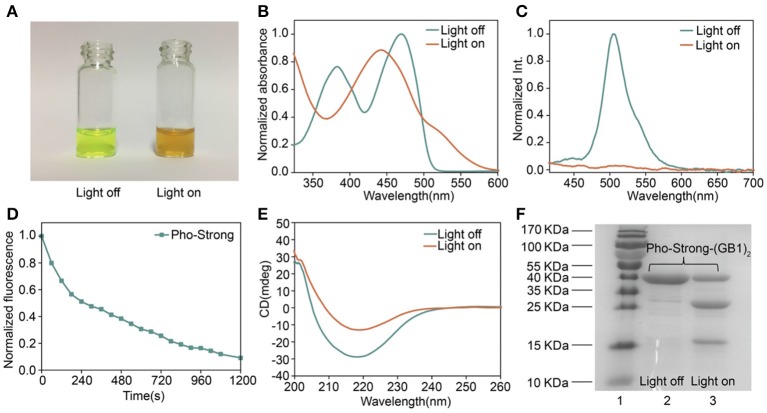
Characterization of Pho-Strong. **(A)** Photographs of Pho-Strong solutions before and after light illumination. **(B)** UV–vis absorbance changes after photocleavage. **(C)** Fluorescence spectra before and after photocleavage excited at 405 nm. **(D)** Kinetics of dissociation, as determined by the decrease of green fluorescence after illumination. **(E)** Circular dichroism spectra before and after photocleavage. **(F)** SDS-PAGE analysis of Pho-Strong-(GB1)_2_ before and after photocleavage. The band at ~ 13 kDa corresponds to the fragment containing (GB1)_2_.

To make sure that the photocleavage properties of the proteins were not affected by the introduction of cysteine mutations, we first characterized their photocleavage properties in solution. The solution of Pho-Weak protein showed characteristic green fluorescence of PhoCl (Figure 3A) (Zhang et al., 2017). After irradiated at 405 nm light for 30 min, the color of Pho-Weak solution changed to brown, consistent with the color change of wild type PhoCl (Zhang et al., 2017). Similarly, UV-vis Spectroscopy of Pho-Weak was also changed and showed a broad absorbance from ~400 to 500 nm after photocleavage (Figure 3B). The fluorescence of Pho-Weak was greatly dimmed after photocleavage (Figure 3C). Using fluorescence change as the indicator, we can estimate the photocleavage kinetics. Pho-Weak was completely cleaved in ~20 min (Figure 3D). The structural change of Pho-Weak was confirmed by the CD spectra. The major negative peak at ~215 nm corresponding to the β sheet structure was greatly reduced after cleavage (Figure 3E). To further confirm that Pho-Weak was cleaved by light illumination, we used SDS-PAGE to characterize the size of photolyzed samples. Because the short fragment is too small to be resolved in SDS-PAGE, we flanked two GB1 proteins as the tag to the C-terminus of Pho-Weak. Clearly, after photocleavage, two additional peaks corresponding to the two fragments appeared (Figure 3F). This indicated that the peptide linkage was completely ruptured. We also performed the characterization of Pho-Strong protein. It showed the same properties as the Pho-Weak protein (Figures 4A–F). The photocleavage kinetics of Pho-Strong was a bit slower than Pho-Weak proteins (t_1/2_ of ~120 s for Pho-Weak and ~270 s for Pho-Strong proteins, respectively). Note that, the photocleavage rates of both Pho-Weak and Pho-Strong proteins were faster than that of wild type PhoCl (t_1/2_ of ~500 s) (Zhang et al., 2017). The photocleavage reaction involves a violet light -induced β-elimination on the conjugated chromophore and the subsequent cleavage of the polypeptide chain. Although the cysteine mutants were distant from the chromophore, they can still affect the photocleavage kinetics. In some of the mutants we tried, the cysteine mutants lost the photocleavage properties because the cysteine mutation sites were at the same loop of the photocleavage site.

### Mechanical Properties of PhoCl-PEG Hydrogel

**Figure 5:**
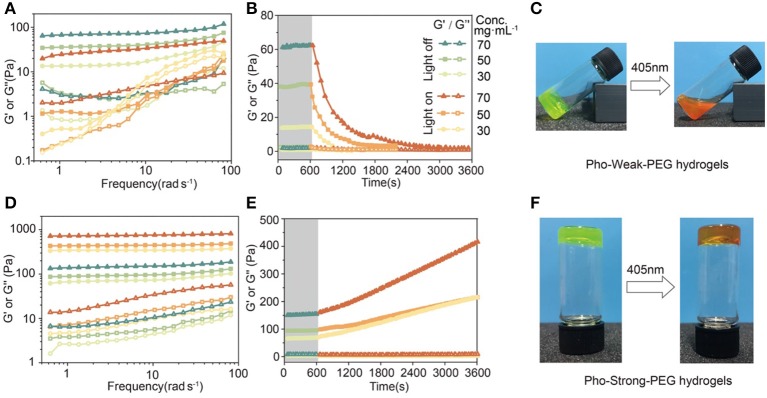
Rheological properties and photo-conversion of PhoCl-PEG hydrogels. **(A)** Frequency sweep of hydrogels with different Pho-Weak concentrations before and after photocleavage. **(B)** Real-time monitoring the photo-weakening of hydrogel by rheology (Frequency: 1 Hz). **(C)** Optical photographs of Pho-Weak-PEG hydrogels: pristine (left, gel) and after photo illumination (right, viscous liquid). **(D)** Frequency sweep of hydrogel with different Pho-Strong concentrations before and after photocleavage. **(E)** Real-time monitoring the photo-weakening of hydrogel by rheology (Frequency: 1 Hz). **(F)** Optical photographs of Pho-Strong-PEG hydrogels: pristine (left) and after photo illumination (right). The legend next to **(B)** applies to **(A, B, D, E)**.

To form the Pho-Weak-PEG hydrogels, Pho-Weak and 4-arm-PEG-Mal were mixed at a molar ratio of 2:1 (solvent exposed thiol:Mal = 1:1) at dark to form Pho-Weak-PEG hydrogel. Thanks to the quick reaction between thiol and maleimide, the hydrogels were formed in <5 min. Rheological measurement showed that the storage modulus G′ of the hydrogels was constantly higher than the loss modulus G″ over a broad frequency range, confirming that a gel network was formed (Figure 5A). With the increase of the protein concentrations, both G′ and G″ increased accordingly. When the hydrogel was irradiated by 405 nm light (468.6 mW·cm^−2^) for 30 min, both G′ and G″ were greatly reduced, confirming the photo-weakening property of the hydrogels. We also monitored the change of the rheological properties of the Pho-Weak-PEG hydrogels at different photo illumination time. A continuous decrease of G′ was observed, indicating that the crosslinking density of the hydrogels were decreased. When 30 mg·mL^−1^ of Pho-Weak was used, the G′ was dropped even lower than G″ after ~1,000 s, indicating that the hydrogel was completely converted to liquid (Figures 5B,C). Note that due to the high concentrations of proteins in the hydrogel, the photocleavage efficiency for Pho-Weak proteins was much lower than that in solution. The efficiency was also inversely correlated with the thickness of the hydrogels.

To form the Pho-Strong-PEG hydrogels, Pho-Strong and 4-armed-PEG-Mal were mixed at a molar ratio of 1:1 (total thiol: Mal = 1:1) at dark to form Pho-Strong-PEG hydrogels, so that parts of PEG arms of the 4-armed-PEG-Mal were still free without participating in the hydrogel network (Figure 2C). When the hydrogels were illuminated at 405 nm light (468.6 mW·cm^−2^) for 40 min, the mechanical properties of the hydrogels became higher (Figure 5D). The constant increase of the mechanical properties of the hydrogels upon photo illumination were observed for the Pho-Strong-PEG hydrogels, further confirming the successful design of the photo-strengthening hydrogels (Figure 5E). Moreover, in the photo-strengthening process, there were no protein aggregation or dehydration effects observed (Figure 5F), suggesting that the increase of the mechanical stability was mainly due to the formation of thiol-maleimide adducts with newly exposed cysteine residues.

It is worth mentioning that both Pho-Weak-PEG and Pho-Strong-PEG hydrogels used in these experiments were not under swelling equilibrium. The swelling of the hydrogels in PBS buffers can also the mechanical properties. The swelling ratios of the Pho-Weak-PEG and Pho-Strong-PEG hydrogels before photolysis were 154 and 196%, respectively, after immersing in PBS buffer for 24 h. Nonetheless, the photo-weakening and photo-strengthening trends of the hydrogels were not affected by the swelling of the hydrogels.

### Spatially Control the Mechanical Properties of PhoCl-PEG Hydrogels

**Figure 6:**
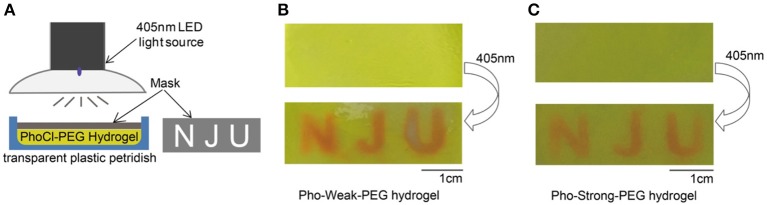
Spatially Control the Mechanical Properties of PhoCl-PEG hydrogels. **(A)** Schematic diagram of the experiment. **(B)** The letters of “NJU” were printed on a Pho-Weak-PEG hydrogel by photo illumination at 405 nm for 40 min through a mask. **(C)** The letters of “NJU” were printed on a Pho-Strong-PEG hydrogel by photo illumination at 405 nm for 40 min through the same mask.

We next explore the use of light to control the mechanical properties of PhoCl-PEG hydrogels in 2D. Both Pho-Weak-PEG and Pho-Strong-PEG hydrogels were prepared in plastic petridishes of ~ 1 mm thick. Then the hydrogels were covered by an alumina foil mask with different patterns (Figure 6A). After illumination under 405 nm light for 40 min, a clear pattern at the light exposed area can be formed (Figures 6B,C). Note that, because the Pho-Weak-PEG hydrogel was partially converted to liquid, the liquidized part was mobile and slowly diffused to the surroundings (Figure 6B). We envisioned that this type of hydrogels can be used for protein/cell delivery in the future. In contrast, the edge of the patterns on the Pho-Strong-PEG hydrogel was much sharper (Figure 6C). It is worth mentioning that as the photocleavage causes the detachment of the C-terminal fragment, peptide or protein release function can be also implement in the Pho-Strong-PEG hydrogels without the dissolution of the hydrogels. This may be even advantageous for long-term on-demand release of functional proteins directly linked to the C-terminal of Pho-Strong protein upon light irradiation. The light convertible mechanical properties also make the hydrogels ideal candidates for the study of the effect of mechanical cues on cell migration, spreading, and differentiation *in vitro*.

## Conclusion

In summary, here we demonstrated the use of a photocleavable protein, PhoCl, as crosslinkers, and multiple-armed PEG as backbones to prepare photo-responsive hydrogels. We engineered two exposed cysteine residues to PhoCl to specifically react with the maleimide group at each end of the PEG arms to form the hydrogels. By choosing proper crosslinking positions, we were able to rationally weakening or strengthening the mechanical properties of the hydrogels upon light illumination. We envision that the release of functional protein/peptide fragments or the expose of bioactive cell binding ligands can be realized simultaneous along with the change of mechanical properties upon light illumination. Therefore, it can be used as an indispensable tool to study the effect of mechanical and chemical cues on stem cell differentiation, cell migration, and so on. Moreover, as the crosslinking density of the hydrogels is greatly changed, it can also be used as a platform for cell capture or cell release. Exploring the biomedical applications of the PhoCl-PEG hydrogels will be our next endeavor.

## Data Availability Statement

The raw data supporting the conclusions of this article will be made available by the authors, without undue reservation, to any qualified researcher.

## Author Contributions

YC, MQ, and WW conceived the idea and supervised the project. YC designed the whole research, and revised the manuscript. DX constructed the plasmids, expressed and purified the proteins, characterized proteins and the hydrogels, and wrote the manuscript. XW and WC were involved in plasmid construction and protein expression. BX helped to prepare and characterize the hydrogels.

### Conflict of Interest

A patent based on this research was filed. The authors declare that the research was conducted in the absence of any commercial or financial relationships that could be construed as a potential conflict of interest.
