# Supplementary material for: Hydrogels With Tunable Mechanical Properties Based on Photocleavable Proteins
Source: Front Chem. 2020 Jan 28;8:7. doi: 10.3389/fchem.2020.00007 (PMC6997547; doi:10.3389/fchem.2020.00007)
Supplement: Supplementary file 1 [file Data_Sheet_1.pdf]

Supplementary Material

Supplementary Figures

	1	2	3	4	5	6	7	8	9	10	11	12	13	14	15	16	17	18	19	20	21	22	23	24	25	26	27	28	29	30
PhoCl	V	I	P	D	Y	F	K	Q	S	F	P	E	G	Y	S	W	E	R	S	M	T	Y	E	D	G	G	I	C	I	A
Pho-Strong	V	I	P	D	Y	F	K	Q	S	F	P	E	G	Y	S	W	E	R	S	M	T	Y	E	D	G	G	I	C	I	A
Pho-Weak	V	I	P	D	Y	F	K	Q	S	F	P	E	G	Y	S	W	E	R	S	M	T	Y	E	D	G	G	I	C	I	A
Pho-Strong-(GB1)$_2$	V	I	P	D	Y	F	K	Q	S	F	P	E	G	Y	S	W	E	R	S	M	T	Y	E	D	G	G	I	C	I	A
Pho-Weak-(GB1)$_2$	V	I	P	D	Y	F	K	Q	S	F	P	E	G	Y	S	W	E	R	S	M	T	Y	E	D	G	G	I	C	I	A

	31	32	33	34	35	36	37	38	39	40	41	42	43	44	45	46	47	48	49	50	51	52	53	54	55	56	57	58	59	60
PhoCl	T	N	D	I	T	M	E	G	D	S	F	I	N	K	I	H	F	K	G	T	N	F	P	P	N	G	P	V	M	Q
Pho-Strong	T	N	D	I	T	M	E	G	C	S	F	I	N	K	I	H	F	K	G	T	N	F	P	C	N	G	P	V	M	Q
Pho-Weak	T	N	D	I	T	M	E	G	D	S	F	I	N	K	I	H	F	K	G	T	N	F	P	C	N	G	P	V	M	Q
Pho-Strong-(GB1)$_2$	T	N	D	I	T	M	E	G	C	S	F	I	N	K	I	H	F	K	G	T	N	F	P	C	N	G	P	V	M	Q
Pho-Weak-(GB1)$_2$	T	N	D	I	T	M	E	G	D	S	F	I	N	K	I	H	F	K	G	T	N	F	P	C	N	G	P	V	M	Q

	61	62	63	64	65	66	67	68	69	70	71	72	73	74	75	76	77	78	79	80	81	82	83	84	85	86	87	88	89	90
PhoCl	K	R	T	V	G	W	E	A	S	T	E	K	M	Y	E	R	D	G	V	L	K	G	D	V	K	M	K	L	L	L
Pho-Strong	K	R	T	V	G	W	E	A	S	T	E	K	M	Y	E	R	D	G	V	L	K	G	D	V	K	M	K	L	L	L
Pho-Weak	K	R	T	V	G	W	E	A	S	T	E	K	M	Y	E	R	D	G	V	L	K	G	D	V	K	M	K	L	L	L
Pho-Strong-(GB1)$_2$	K	R	T	V	G	W	E	A	S	T	E	K	M	Y	E	R	D	G	V	L	K	G	D	V	K	M	K	L	L	L
Pho-Weak-(GB1)$_2$	K	R	T	V	G	W	E	A	S	T	E	K	M	Y	E	R	D	G	V	L	K	G	D	V	K	M	K	L	L	L

	91	92	93	94	95	96	97	98	99	100	101	102	103	104	105	106	107	108	109	110	111	112	113	114	115	116	117	118	119	120
PhoCl	K	G	G	G	H	Y	R	C	D	Y	R	T	T	Y	K	V	K	Q	K	P	V	K	L	P	D	Y	H	F	V	D
Pho-Strong	K	G	G	G	H	Y	R	C	D	Y	R	T	T	Y	K	V	K	Q	K	P	V	K	L	P	D	Y	H	F	V	D
Pho-Weak	K	G	G	G	H	Y	R	C	D	Y	R	T	T	Y	K	V	K	Q	K	P	V	K	L	P	D	Y	H	F	V	D
Pho-Strong-(GB1)$_2$	K	G	G	G	H	Y	R	C	D	Y	R	T	T	Y	K	V	K	Q	K	P	V	K	L	P	D	Y	H	F	V	D
Pho-Weak-(GB1)$_2$	K	G	G	G	H	Y	R	C	D	Y	R	T	T	Y	K	V	K	Q	K	P	V	K	L	P	D	Y	H	F	V	D

	121	122	123	124	125	126	127	128	129	130	131	132	133	134	135	136	137	138	139	140	141	142	143	144	145	146	147	148	149	150
PhoCl	H	R	I	E	I	L	S	H	D	K	D	Y	N	K	V	K	L	Y	E	H	A	V	A	R	N	S	T	D	S	M
Pho-Strong	H	R	I	E	I	L	S	H	D	K	D	Y	N	K	V	K	L	Y	E	H	A	V	A	R	N	S	T	D	S	M
Pho-Weak	H	R	I	E	I	L	S	H	D	K	D	Y	N	K	V	K	L	Y	E	H	A	V	A	R	N	S	T	D	S	M
Pho-Strong-(GB1)$_2$	H	R	I	E	I	L	S	H	D	K	D	Y	N	K	V	K	L	Y	E	H	A	V	A	R	N	S	T	D	S	M
Pho-Weak-(GB1)$_2$	H	R	I	E	I	L	S	H	D	K	D	Y	N	K	V	K	L	Y	E	H	A	V	A	R	N	S	T	D	S	M

	151	152	153	154	155	156	157	158	159	160	161	162	163	164	165	166	167	168	169	170	171	172	173	174	175	176	177	178	179	180
PhoCl	D	E	L	Y	K	G	G	S	G	G	M	V	S	K	G	E	E	T	I	T	S	V	I	K	P	D	M	K	N	K
Pho-Strong	D	E	L	Y	K	G	G	S	G	G	M	V	S	K	G	E	E	T	I	T	S	V	I	K	P	D	M	K	N	K
Pho-Weak	D	E	L	Y	K	G	G	S	G	G	M	V	S	K	G	E	E	T	I	T	S	V	I	K	P	D	M	K	N	K
Pho-Strong-(GB1)$_2$	D	E	L	Y	K	G	G	S	G	G	M	V	S	K	G	E	E	T	I	T	S	V	I	K	P	D	M	K	N	K
Pho-Weak-(GB1)$_2$	D	E	L	Y	K	G	G	S	G	G	M	V	S	K	G	E	E	T	I	T	S	V	I	K	P	D	M	K	N	K

	181	182	183	184	185	186	187	188	189	190	191	192	193	194	195	196	197	198	199	200	201	202	203	204	205	206	207	208	209	210
PhoCl	L	R	M	E	G	N	V	N	G	H	A	F	V	I	E	G	E	G	S	G	K	P	F	E	G	I	Q	T	I	D
Pho-Strong	L	R	M	E	G	N	V	N	G	H	A	F	V	I	E	G	E	G	S	G	K	P	F	E	G	I	Q	T	I	D
Pho-Weak	L	R	M	E	G	N	V	N	G	H	A	F	V	I	E	G	E	G	S	G	K	P	F	E	G	I	Q	T	I	D
Pho-Strong-(GB1)$_2$	L	R	M	E	G	N	V	N	G	H	A	F	V	I	E	G	E	G	S	G	K	P	F	E	G	I	Q	T	I	D
Pho-Weak-(GB1)$_2$	L	R	M	E	G	N	V	N	G	H	A	F	V	I	E	G	E	G	S	G	K	P	F	E	G	I	Q	T	I	D

	211	212	213	214	215	216	217	218	219	220	221	222	223	224	225	226	227	228	229	230	231	232	233	234	235	236	237	238	239	240
PhoCl	L	E	V	K	E	G	A	P	L	P	F	A	Y	D	I	L	T	T	A	F	H	Y	G	N	R	V	F	T	K	Y
Pho-Strong	L	E	V	K	E	G	A	P	L	P	F	A	Y	D	I	L	T	T	A	F	H	Y	G	N	R	V	F	T	K	Y
Pho-Weak	L	E	V	K	E	G	A	P	L	P	F	A	Y	D	I	L	T	T	A	F	H	Y	G	N	R	V	F	T	K	Y
Pho-Strong-(GB1)$_2$	L	E	V	K	E	G	A	P	L	P	F	A	Y	D	I	L	T	T	A	F	H	Y	G	N	R	V	F	T	K	Y
Pho-Weak-(GB1)$_2$	L	E	V	K	E	G	A	P	L	P	F	A	Y	D	I	L	T	T	A	F	H	Y	G	N	R	V	F	T	K	Y

	241	242	243	244	245	246	247	248	249	250	251	252	253	254	255	256	257	258	259	260	261	262	263	264	265	266	267	268	269	270
PhoCl	P	R																												
Pho-Strong	P	R																												
Pho-Weak	P	R	G	G	G	R	S	G	G	C																				
Pho-Strong-(GB1)$_2$	P	R	G	G	G	R	S	M	D	T	Y	K	L	I	L	N	G	K	T	L	K	G	E	T	T	T	E	A	V	D
Pho-Weak-(GB1)$_2$	P	R	G	G	G	R	S	M	D	T	Y	K	L	I	L	N	G	K	T	L	K	G	E	T	T	T	E	A	V	D

	271	272	273	274	275	276	277	278	279	280	281	282	283	284	285	286	287	288	289	290	291	292	293	294	295	296	297	298	299	300
PhoCl																														
Pho-Strong																														
Pho-Weak																														
Pho-Strong-(GB1)$_2$	A	A	T	A	E	K	V	F	K	Q	Y	A	N	D	N	G	V	D	G	E	W	T	Y	D	D	A	T	K	T	F
Pho-Weak-(GB1)$_2$	A	A	T	A	E	K	V	F	K	Q	Y	A	N	D	N	G	V	D	G	E	W	T	Y	D	D	A	T	K	T	F

	301	302	303	304	305	306	307	308	309	310	311	312	313	314	315	316	317	318	319	320	321	322	323	324	325	326	327	328	329	330
PhoCl																														
Pho-Strong																														
Pho-Weak																														
Pho-Strong-(GB1)$_2$	T	V	T	E	R	S	M	D	T	Y	K	L	I	L	N	G	K	T	L	K	G	E	T	T	T	E	A	V	D	A
Pho-Weak-(GB1)$_2$	T	V	T	E	R	S	M	D	T	Y	K	L	I	L	N	G	K	T	L	K	G	E	T	T	T	E	A	V	D	A

	331	332	333	334	335	336	337	338	339	340	341	342	343	344	345	346	347	348	349	350	351	352	353	354	355	356	357	358	359	360
PhoCl																														
Pho-Strong																														
Pho-Weak																														
Pho-Strong-(GB1)$_2$	A	T	A	E	K	V	F	K	Q	Y	A	N	D	N	G	V	D	G	E	W	T	Y	D	D	A	T	K	T	F	T
Pho-Weak-(GB1)$_2$	A	T	A	E	K	V	F	K	Q	Y	A	N	D	N	G	V	D	G	E	W	T	Y	D	D	A	T	K	T	F	T

	361	362	363	364	365
PhoCl					
Pho-Strong					
Pho-Weak					
Pho-Strong-(GB1)$_2$	V	T	E	R	S
Pho-Weak-(GB1)$_2$	V	T	E	R	S

Supplementary Figure 1. Sequence alignment of PhoCl and PhoCl variants. Mutations relative to PhoCl are represented highlighted with a green background. GB1 fragments are highlighted by a cyan background. The site of photocleavage is indicated with an arrow and purple background.

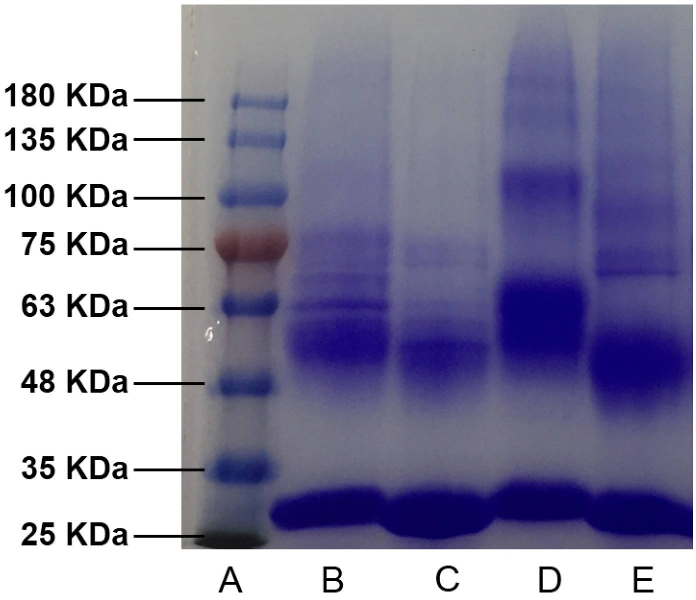

Supplementary Figure 2. SDS-PAGE of PhoCl mutants reacting with PEG-Mal (10 kDa) before and after photocleavage. A: Ladder; B: Pho-Weak Light off; C: Pho-Weak Light on; D: Pho-Strong Light off; E: Pho-Strong Light on. Based on the grey scale analysis, the reaction yields were 54% for Pho-Weak and 65% for Pho-Strong. The success of the photocleavage reaction was also confirmed.
